# Influence of Thermal Treatment on the Composition of *Alpinia officinarum* Rhizome

**DOI:** 10.3390/ijms25073625

**Published:** 2024-03-24

**Authors:** Justyna Zagórska, Karolina Pietrzak, Wirginia Kukula-Koch, Marcin Czop, Karolina Wojtysiak, Wojciech Koch

**Affiliations:** 1 Department of Food and Nutrition, Medical University of Lublin, 4a Chodzki Str., 20-093 Lublin, Poland; justyna.zagorska@umlub.pl (J.Z.); karolinapietrzak94@gmail.com (K.P.); karolinawojtysiak17@gmail.com (K.W.); 2 Department of Pharmacognosy with Medical Plants Garden, Medical University of Lublin, 1 Chodzki Str., 20-093 Lublin, Poland; virginia.kukula@gmail.com; 3 Department of Clinical Genetics, Medical University of Lublin, 11 Radziwiłłowska Str., 20-080 Lublin, Poland; marcin.czop@umlub.pl

**Keywords:** *Alpinia officinarum*, Zingiberaceae, thermal treatment, HPLC-MS, HPLC, DPPH, Folin–Ciocalteu, polyphenols

## Abstract

*Alpinia officinarum* is a representative of the Zingiberaceae family, which is known for its wide use in the food and pharmaceutical industries also due to its precious pharmacological potential. The major aim of the present study was to evaluate the influence of thermal treatment on the composition of the rhizome of *Alpinia officinarum* and its antioxidant activity. The fresh rhizome was subjected to various thermal treatment processes—boiling, frying and microwave heating during various time intervals—and their composition and antioxidant activity were determined using chromatographic (HPLC – High Performance Liquid Chromatography and HPLC-MS - High Performance Liquid Chromatography Mass Spectrometry) and spectrophotometric (DPPH and TPC – Total Phenolic Content) methods. Pinobanksin was the main compound found in the extract of the fresh rhizome (537.79 mg/kg), followed by galangin (197.7 mg/kg) and zingerone (185.5 mg/kg). The effect of thermal treatment on the rhizome composition was varied. In general, thermal processing significantly decreased the content of active compounds in the rhizome. However, there were some exceptions—boiling for 4 min significantly increased the content of pinobanksin (1162.4 mg/kg) and galangin (280.7 mg/kg), and microwave processing for 4 min increased the content of pinocembrin (213 mg/kg). It was found that boiling and microwave treatment significantly increased the antioxidant activity of the processed rhizomes.

## 1. Introduction

For many centuries, plants and their extracts have been used not only as a source of food, but also to treat diseases and support basic body functions. People gained knowledge about how to use the medicinal plants in pathological conditions, their pharmacological properties and possible side effects from their own experiences and observations. Phytotherapy is considered as a safer, less toxic and lower-cost approach in comparison to classical medicine which uses synthetic drugs. So far, much research has been carried out to determine the structures of chemical compounds responsible for the therapeutic effect of natural products, which confirmed their healing effects and also enabled further work on new applications and the use of plant raw materials. Herbal medicine is the basis of health care, especially in underdeveloped countries, while herbal medicines have recently become more and more popular also in developed countries due to the growing public awareness of the importance of diet in maintaining health and good condition of the body [1].

Natural drugs are more easily available. Also, multi-ingredient drugs are often a better choice especially when considering antimicrobial effects or anticancer therapies as they are more effective despite the developed resistance to single-ingredient drugs [1,2]. 

Zingiberaceae is one of the most widespread botanical families that is abundantly spread around African, Asian and American countries. It comprises many species of plants with valuable therapeutic and nutritional properties and have various pharmacological and biological activities, including antiseptic, antiallergic and antipruritic properties [3,4,5,6,7,8,9,10,11]. Most of them are used as medicinal products, perfume additives, flavors, dyes and spices. One of the main recognized Zingiberaceae genera is *Alpinia* with its wide range of medical and non-medical applications. *A. officinarum*, *A. zerumbet* and *A. oxyphylla* are the best studied species. The former has recently gained in importance in the food and pharmaceutical industries [12]. In the literature, *Alpinia officinarum* is also called lesser galangal. The plant comes from China and Indochina, but is widely cultivated in the plains of West Bengal and Southeast Asian countries, for example in Vietnam [13], where tropical conditions, high humidity and temperatures exceeding 15 °C positively influence the cultivation of this plant [14]. The mineral composition of the rhizome of *Alpinia officinarum* includes a high content of K, Mg and Ca, as well as lower concentrations of Na, Mn or Fe. Its general organic composition consists of approximately 20% tannins, 18% proteins, 17% carbohydrates, 15% lipids, 5% flavonoids and 3% phenolic compounds [12].

For hundreds of years, dried rhizomes of galangal have been used as an ingredient of diet, but also as a medicinal plant, used in traditional Chinese medicine due to its multi-directional therapeutic effects, namely improving digestion and relieving the symptoms of colds, inflammation, abdominal pain, diarrhea and stomach ulcers. Due to its strong, aromatic smell and spicy taste, the rhizome of galangal is used in Asian cuisine as a food spice [15,16].

The rhizome of *Alpinia officinarum* is a rich source of active ingredients with medicinal properties. About 90 biologically active ingredients have been isolated and identified from it, containing mainly phenolic compounds, especially diarylheptanoids, which are one of the most efficient active substances [14]. Another important compound found in *Alpinia* rhizome is galangin (3,5,7-trihydroxyflavone), one of the polyphenolic active compounds present in *Alpinia officinarum* and honey, belonging to flavonoids [17,18]. Flavonoids have a multidirectional therapeutic effect, which has been proven in many in vivo and in vitro studies. Some studies proved anticancer activity of galangin [19,20]. In the methanolic extract of the rhizome of *Alpinia officinarum*, nine glycoside compounds were identified and isolated, which were described to have biological activity [21]. The next group of biologically active compounds found in the rhizome of *Alpinia* is terpenes, which constitute one of the largest and most diverse natural groups of active compounds [22]. The main constituents of the essential oil from the plants of the Zingiberaceae family are α-terpinyl, β-turmerone, α-zingiberene and 1,8-cineole [23]. The biological activities of the extracts from *Alpinia officinarum* depend also on the method of extraction. It was proved that aqueous extracts have the lowest biological activity compared to extracts obtained using organic solvents (methanol and ethanol) [13,23].

Many studies on the biological properties of *Alpinia officinarum* indicated its antioxidant [23,24], antiviral [25,26], antibacterial [27,28], antifungal [29,30], antimicrobial [31], analgesic and anti-inflammatory effects [32,33]. Aqueous extracts have also been studied in skin problems [34], showing antiphotoaging effects [35] and in studies on the pharmacological activity and therapeutic potential of galangin in age-related diseases [36]. Moreover, *Alpinia* has also been shown to have beneficial effects on the digestive [37,38,39,40,41]), respiratory [42,43], nervous [44,45,46,47,48] and circulatory systems [49] and also on bone structure [50,51]. The biological activities of *Alpinia* rhizome also include antidiabetic effect (especially type 2 diabetes) [52,53,54,55], inhibition of pancreatic lipase and antihyperlipidemic effect, lowering blood cholesterol [56], as well as helping to regulate body weight, which is important in the treatment of obesity [57,58]. Research on the anti-genotoxicity properties of galangal and its possible use as a cancer chemopreventive agent is also particularly developed [59]. Studies on the prevention and treatment of various types of cancer have been described: esophagus [60], liver [61,62,63], breast [64], lungs [65] or gastric cancer [66].

The multitude of various research on the effects of *Alpinia officinarum* extracts on the body confirms the importance of studies on efficient methods of extraction to obtain rich extracts in terms of its qualitative and quantitative composition, but also depending on the quality of the plant material and the method of processing before obtaining a final extract. The subject of this study was to investigate the impact of thermal treatment on the content of active ingredients present in the rhizome of *Alpinia officinarum*, depending on the applied processing method and time, which are two the most important parameters in the process of preparing dishes. The study used a fresh rhizome of *Alpinia officinarum*, which was fried, boiled and microwaved for 1, 2, 4 and 10 min. These three main types of processing were chosen as they are the most frequently used during cooking and preparing meals. Changes in the content of the main active compounds and antioxidant activity of the samples were assessed using the HPLC and spectrophotometric methods used to evaluate antioxidant activity of the samples—TPC using Folin–Ciocalteu reagent and the DPPH scavenging radical assay. The research hypothesis assumed that thermal treatment would cause a decrease in the content of the main active constituents present in the rhizome, which would result in a reduction in the antioxidant activity of the studied extracts. To the best of our knowledge, it is the first study in which a thorough analysis of the changes in composition of secondary metabolites of *Alpinia officinarum* rhizome and its antioxidant properties due to various thermal processing methods was evaluated, using modern chromatographic and spectrophotometric techniques.

## 2. Results

### 2.1. The Fingerprinting of Extracts by HPLC-ESI-QTOF-MS/MS Method

**Figure 1:**
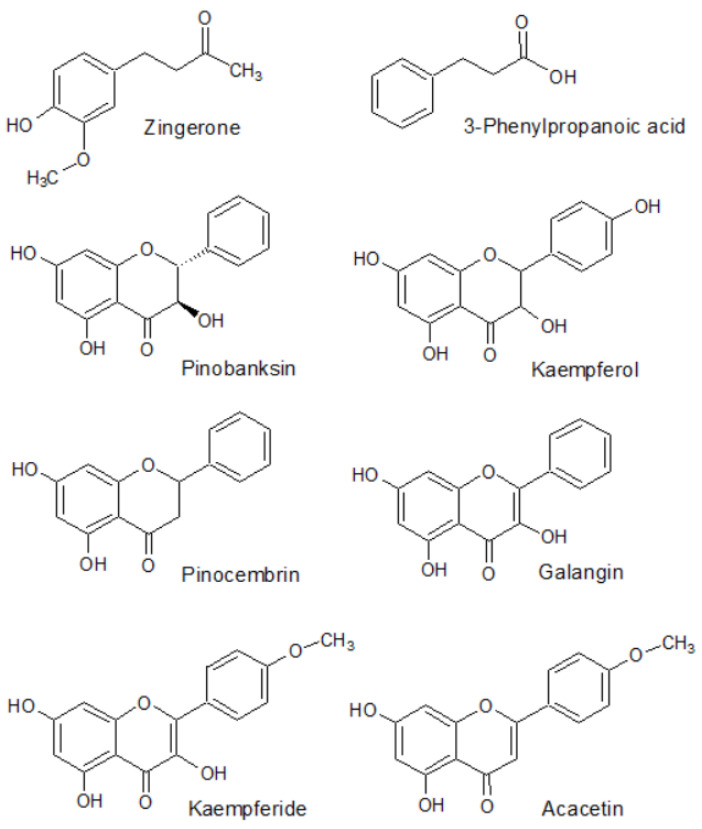
Structural formulas of chemical compounds present in the rhizome of *Alpinia officinarum* identified and determined using chromatographic methods.

The HPLC-MS method enabled the identification of eight active constituents present in the rhizome of *Alpinia officinarum*—zingerone, 3-phenylpropanoic acid, pinobanksin, kaempferol, pinocembrin, galangin, kaempferide and acacetin as the major components of the extract for which the quantitative analysis could be performed afterwards. The structural formulas of these compounds are presented in Figure 1. Table 1 presents the parameters which were used during the identification of these compounds using the mass spectrometry technique. The MS/MS spectra that were used for the identification of the major metabolites are presented in the Supplementary Materials (Table S1).

The main metabolites present in the mass chromatograms belonged to the group of flavonoids. *Alpinia officinarum* was proved to contain kaempferol and pinobanksin as the representatives of trihydroxyflavanones, a flavanone—pinocembrin, galangin and kaempferide—two flavonols, and acacetin from the group of flavones. The list is completed by two structures: zingerone, a derivative of vanillylacetone, and 3-phenylpropanoic acid. Previously, the compounds were identified in the same plant genus by other authors and were perceived as the molecules responsible for the pharmacological potential of the total extracts [67,68].

For zingerone, we have observed a clear molecular peak at 10.01 min with *m/z* of 193.0870 that corresponded to the formula C_11_H_14_O_3_. The fragmentation of this compound was scarce. It provided only a prominent fragmentation ion of 179.0030 Da. It can be formed by the loss of the methyl group at the methoxy substituent of the ring. 3-Phenylpropanoic acid whose *m/z* signal of 149.06080 Da was observed also in the negative ionization mode in the 12.3 min decomposed to a 105.0556 Da fragment ion by the loss of an acetyl group. The following compounds from Table 1 are flavonoids and they undergo a typical fragmentation pattern. For pinobanksin, the major component of the tested extract that was eluted from the column in the 15th min with the molecular ion of 271.0612 Da, several fragment ions were recorded in the MS/MS spectrum. Among them, the most important fragmentation ones were the fragment ion with the *m/z* of 253.0509 Da that formed due to the loss of water and with the *m/z* of 151.0041 Da due to the cleavage of the C ring of flavonol [69]. For kaempferol (C_15_H_10_O_6_), at 16.4 min two fragment ions were recorded in the collision energy of 20 V: 257.0453 and 229.0495 Da that came from the C_14_H_9_O_5_^+^ and C_13_H_9_O_4_^+^ structures, respectively, that were previously identified by March and Miao [70]. A flavanone, pinocembrin, was characterized by an accurate mass of 255.0663 u at 22.4 min that showed 213.0594 (26.34%) and 151.0004 (24.7%) *m/z* fragment ions in the MS/MS spectrum which appeared from the opening of the flavonoid ring giving the formulas (C_13_H_9_O_3_)− and (C_7_H_3_O_4_)−, respectively [69]. For galangin, the MS/MS spectra showed three fragment ions with *m/z* 227.0360 (1.22%), 213.0564 (2.22%) and 197.0619 (1.79%). The former and the latter are obtained by a successive loss of CO_2_ and CO groups. The middle fragment appeared after the detachment of two CO moieties from the molecular ion [71]. In the case of kaempferide (C_16_H_12_O_6_), eluted in the 22.9 min as an *m/z* of 299.0561, the loss of a hydroxyl group was observed (*m/z* of 284.0318) together with the Diels–Alder reaction of flavonoid ring fragmentation (*m/z* of 151.0029) [71]. Finally, the MS/MS spectrum of acacetin (C_16_H_12_O_5_) at 23.8 min was analyzed with the most prominent fragment ions at 268.0391(100%) and 239.0359 (96.2%) Da coming from the molecular ion of 283.0612 Da. The 268 fragment ion came from the detachment of a methyl group at the phenolic ring, whereas the 239 signal was a result of the Diels–Alder reaction and opening of the ring B [72], similarly to other flavonoids.

### 2.2. Quantitative Analysis of Identified Compounds Using HPLC Method

**Figure 2:**
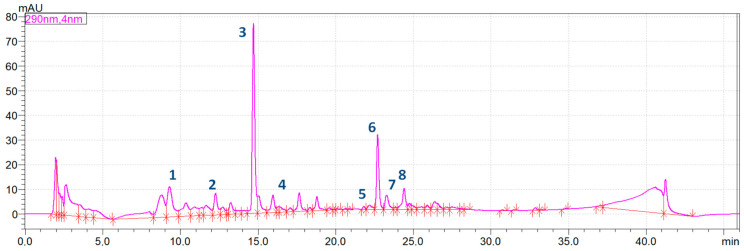
The integrated HPLC chromatogram of *Alpinia officinarum* fresh rhizome extract (1—zingerone, 2–3-phenylpropanoic acid, 3—pinobanksin, 4—kaempferol, 5—pinocembrin, 6—galangin, 7—kaempferide, 8—acacetin).

All active constituents which were identified in the extracts from the rhizome of *Alpinia officinarum* by HPLC-MS were quantitatively determined using the HPLC method with DAD detection. The calculated contents of compounds in *Alpinia* extracts depending on the type of thermal treatment are presented in Table 2. Figure 2 shows the HPLC chromatogram obtained for the extract of fresh, unprocessed rhizome. HPLC chromatograms recorded for the analysed samples that undergone the thermal processing by boiling, frying and microwave heating were presented in Figure S1 in the Supplementary Materials.

The obtained results show that the type of culinary process significantly affects the content of active compounds. 

The analysis of *Alpinia officinarum* rhizome extracts using HPLC allowed for the quantitative determination of eight compounds, which were: zingerone, 3-phenylpropanoic acid, pinobanksin, kaempferol, pinocembrin, galangin, kaempferide and acacetin. The compounds were identified by comparing the retention times of the peaks formed in the chromatograms to those obtained with the same method parameters using LC-MS. 6-gingerol, the compound of the same chemical nature and with a similar UV spectrum and maximum absorbance at 290 nm as the identified active substances, was used as an external standard for quantitative determinations. Previous studies of this plant confirmed the presence of flavonoids in the rhizome of *Alpinia officinarum*: galangin, kaempferol and quercetin [73]. Based on the obtained results, it was shown that pinobanksin was by far the major compound present in the rhizome of *Alpinia officinarum*, the content of which in the fresh rhizome was almost 538 mg/kg. Its content changed under the influence of both the type and time of thermal treatment. Boiling was a thermal process which has significantly increased the concentration of pinobanksin in the studied rhizome and the obtained results revealed that the highest concentration was determined in samples boiled for 4 min—1162 mg/kg, which was almost twice as much as the fresh rhizome. On the other hand, frying significantly decreased the concentration of pinobanksin and the lowest concentration was observed in samples fried for 1 min—44.3 mg/kg. Galangin was the second major active constituent, the content of which in the fresh rhizome was almost 198 mg/kg. Similar to pinobanksin, almost all thermal processing methods significantly reduced its content, except boiling for 4 min, which significantly increased its content in the rhizome (280 mg/kg). Zingerone was the third major compound identified in the studied extracts; however, thermal processing significantly decreased its concentration in almost all processed samples, except microwave treatment for 4 min, which slightly increased its content, but this change was insignificant. The content of other active compounds identified in the rhizome was below 100 mg/kg and applied thermal processing in most cases significantly reduced its content in the extract. However, there were some exceptions—microwave treatment for 4 min increased (insignificantly) the content of 3-phenylpropanoid acid (88 mg/kg); boiling performed for 1, 2 and 4 min significantly increased the content of kaempferol in comparison to fresh rhizome; boiling for 1 min increased insignificantly the content of pinocembrin and 4 min boiling, frying and especially microwave processing significantly enhanced it; boiling for 1 and 4 min and microwave processing for 4 min significantly increased the concentration of kaempferide. Interestingly, it was revealed that acacetin was the only active constituent present in the rhizome of *Alpinia officinarum* for which all thermal methods significantly decreased its content in comparison to the fresh material. Comparing all types and times of thermal treatment, two methods seem to be the most promising as the methods allowing to obtain the highest content of the determined compounds. The first one is microwave heating for 4 min, which caused the highest amount of zingerone—188.59 mg/kg; 3-phenylpropanoic acid—87.64 mg/kg; pinocembrin—212.94 mg/kg and kaempferide—79.22 mg/kg, while the second one is boiling, also for 4 min, which resulted in the highest amounts of pinobanksin—1162.40 mg/kg and galangin—280.69 mg/kg. In turn, the highest kaempferol content was recorded for the sample boiled for 1 min—85.56 mg/kg.

### 2.3. Influence of Thermal Processing on the Antioxidant Activity of Alpinia officinarum

The results obtained using the DPPH radical (Table 3) showed that boiling the rhizome for 1, 2, 4 and 10 min, as well as exposure to microwaves for 1, 2 and 4 min, resulted in a significant increase in the radical scavenging activity of the samples compared to the fresh raw material. Thermal processing using microwaves for 2 and 4 min resulted in a slightly lower increase in the antioxidant activity compared to this type of treatment lasting 1 min. In the case of frying, only a slight increase was observed for 1 and 2 min, but these changes were insignificant, while for the longer process lasting 4 and 10 min there was a decrease in the potential against free radicals, although only the first change was considered significant. Comparing the obtained data for all samples, it can be seen that the rhizome boiled for 10 min was characterized by the highest antioxidant activity, 81.78%. In contrast, the activity to deactivate DPPH radicals of the samples which were fried for 4 min was only 21.60%. On this basis, it can be seen that, depending on the type of thermal treatment and its duration, the activity against free radicals can changed almost four times.

The results of tests using the Folin–Ciocalteu reagent (Table 3) were comparable to those obtained using the DPPH radical. Again, the frying process was one that showed the most deteriorating effect on the antioxidant activity of *Alpinia* rhizomes. It was frying which caused the highest decrease in antioxidant activity, which was correlated with frying time—the longer the time, the higher the reduction in antioxidant activity. The thermal process of the *Alpinia* rhizome, consisting of boiling or the use of microwaves, resulted in a significant increase in the total polyphenol content (TPC) compared to the content of these compounds in the unprocessed *Alpinia* rhizome which was 128.32 mg GAE/L.

Both the total polyphenol content and the scavenging power of the DPPH radical testify to the antioxidant potential of the tested sample. When comparing the results obtained using the Folin–Ciocalteu method and the DPPH test, some similarities can be noticed. In both methods, comparing all types of heat treatment, the lowest values were determined for fried samples. On the other hand, comparing the duration of frying in both cases, subjecting the rhizome to this process for a short time—1 or 2 min—was more beneficial than extending the treatment to 4 or 10 min. Boiling contributed to an increase in both parameters compared to the fresh rhizome. Comparing the effect of processing time in both cases, boiling for 4 min turned out to be the least beneficial. Interestingly, the largest differences in the effect of treatment on TPC and DPPH radical scavenging percentage were noted for the microwave processing method. It was found that the longest of the studied microwave treatment times (4 min) gave the highest results in TPC test, while the shortest (1 min) contributed to the highest results obtained in the DPPH assay.

### 2.4. Correlation between Compound Content and Antioxidant Activity

In a further step, correlation analysis was performed between the content of the tested compounds and antioxidant activity (Table 4). Correlating the results obtained using the Folin–Ciocalteu reagent, positive correlations were found with the content of the following compounds: 3-phenylpropanoic acid (r = 0.54; *p* < 0.001), pinobanksin (r = 0.36; *p* < 0.05), kaempferol (r = 0.47; *p* < 0.01), kaempferide (r = 0.57; *p* < 0.001). The above results suggest that the aforementioned compounds have the greatest relationship with the activity associated with the use of Folin–Ciocalteu reagent.

In addition, the results of this analysis show a positive correlation between the results of the analysis using the DPPH radical and the content of the following compounds: pinobanksin (r = 0.61; *p* < 0.001), kaempferol (r = 0.51; *p* < 0.01) (Table 4). These results indicate that these compounds, i.e., pinobanksin and kaempferol, have the greatest relationship with the activity associated with the use of the DPPH radical.

It can be concluded, that based on the obtained results, it seems that pinobanksin and kaempferol have the most significant influence on the antioxidant activity of the rhizome of *Alpinia officinarum*. 

## 3. Discussion

Most culinary dishes are prepared after a previously used technological process. Such processes include, among others: boiling, steaming, frying, baking, grilling, stewing or heating with microwaves. The use of thermal treatment is intended to ensure appropriate quality and give the desired organoleptic characteristics to the prepared meals. In addition, it removes impurities and microorganisms, and increases the absorption of nutrients [74]. Unfortunately, exposing food products to high temperatures may also cause some loss of nutrients. These losses may depend, for example, on the heating method used and the process temperature [75]. Research has shown that the greatest losses are observed after long-term boiling without reusing the stock and during long-term frying [76]. Most of the research conducted so far has focused on the impact of thermal processes on the content of nutrients, while much less is known about the influence on non-nutrients, which are important active compounds, which are part of various plants used as medicines and food [76,77,78]. Before thermal processing, the food product should be subjected to preliminary stages, i.e., washing, drying and grinding. These processes ensure the removal of impurities, pathogens and inedible parts, and also gives it the appropriate form. During preliminary processing, an intermediate product can be obtained, that must then be subjected to further processing [77]. Boiling is the most common method of thermal treatment. Vegetables, fruits, meat and eggs are most often prepared this way. The procedure involves exposing food products to hot water or steam at a temperature of approximately 100 °C. Foods prepared by boiling can lose 30 to 50% of their nutrients [79,80,81,82]. Water-soluble vitamins and phenolic compounds are particularly sensitive to this type of heat treatment. To reduce losses, preparing the meals in the shortest possible time and with the least amount of water, and using the resulting stock for soups or sauces is recommended [77,78,83]. Frying is another popular method of thermal treatment. This process takes place in the temperature range from 130 to 220 °C. Depending on the temperature, fat-free frying, frying with a thin layer of fat and deep fat can be distinguished. During frying, complex reactions occur between the food matrix and the fat used. The process inactivates enzymes, reduces the water content in food and destroys microorganisms [84,85,86]. Long-term exposure to high temperatures adversely affects the content of nutrients such as vitamins, peptides and antioxidants [78,83]. Too frequent consumption of fried foods carries the risk of developing metabolic diseases—atherosclerosis, obesity, diabetes and many others [87,88]. Thus, this type of food processing is currently considered as the most negative towards human health. Heating food with microwaves uses electromagnetic radiation with a vibration frequency of 915 and 2450 MHz. Microwaves set in motion dipole water molecules, which collide with ions in the electromagnetic field and cause friction. Friction produces heat that warms the product [89]. Research on the effect of microwave heating on the content of nutrients showed that vegetable protein exposed to microwaves had better digestibility compared to raw protein [90,91]. Additionally, smaller losses of protein contained in fish were observed during microwave defrosting [92,93,94]. No significant differences in changes in the content of microwave-processed and traditional carbohydrates were documented. In the case of vitamins, performed studies revealed that a short heat treatment time results in smaller vitamin losses compared to other methods [95]. However, it was also found that not only the selection of food products for the daily diet but also the methods of their thermal treatment have a significant impact on, among other things, the speed of skin aging processes. Even a list of recommendations was created, including limiting unnecessary thermal processes or shortening their duration to the necessary minimum, as well as adding ingredients with anti-glycation properties (such as ginger, turmeric, cinnamon, cloves, garlic and other green herbs). Because these recommendations are quite general, this type of research must be continued and developed to provide more details and guidance on how to eat to obtain the most benefits from the foods we choose [96].

The Zingiberaceae family, which includes *Zingiber officinale*, *Curcuma longa* and *Alpinia officinarum*, is known for its high content of compounds with anti-inflammatory, neuroprotective, hepatoprotective [97], antidiabetic, anticancer or antioxidant properties. These plants are rich in a number of different compounds, responsible for, among other things, their taste, smell and medicinal properties. Thanks to the development of analytical methods, it is possible to determine hundreds of them using GC-MS and LC-MS techniques [98]. Among them, the most frequently researched compounds with antiradical activity include curcuminoids and gingerols [24]. The most widely known plants from this family are ginger and turmeric. Research has proven that both ginger and its products can be successfully used as antitussives, antiemetics and to provide relief from digestive disorders. This is mainly due to the polyphenol and terpene compounds found in the rhizome [99]. In turn, curcuminoids present in turmeric rhizome prevent cancer and cardiovascular diseases [100]. As was mentioned above, there are no publications in the literature focusing on the impact of thermal treatment on the composition of *Alpinia officinarum* rhizome. Research on ginger and turmeric is much more developed, where significant number of articles as well as review manuscript summarizing the available research results can be found. Gunasena et al. studied, among other things, the effect of boiling and its time on the antioxidant and antimicrobial activity of ginger and turmeric, but also cinnamon and garlic. By measuring the content of malonaldehyde (MDA) depending on the temperature and duration of the boiling process, it was found that with prolonged boiling of the raw material, the antioxidant activity decreased (a greater decrease in the case of turmeric in comparison to ginger was observed), and ginger itself showed better antioxidant properties than turmeric [101]. Koch et al. drew similar conclusions regarding the reduction in antiradical potential of ginger extract as a result of long-term boiling, using the IC_50_ parameter, which is negatively correlated with antiradical properties using DPPH assay. In this study, the IC_50_ value for boiling for 5 min increased from 235 to 340 µg/mL. In the case of frying, the difference was even greater—300 to 940 µg/mL (for 2 and 15 min, respectively). However, the opposite tendency was observed for microwave heating, where the IC_50_ decreased from 200 to 150 µg/mL (for 1 and 5 min, respectively) while the value for fresh ginger rhizome was 210 µg/mL, which showed a significant increase in the antiradical activity [102]. An et al. found that microwave drying of *Zingiber officinale* rhizome resulted in a decrease in TPC and had almost no effect on antioxidant potential compared to fresh ginger extract (DPPH, FRAP, ABTS tests) [103]. On the other hand, Kim et al., studying the effect of microwave radiation on the antioxidant activity of ginger rhizome extract, noticed a negative effect of this type of treatment (DPPH test) [104]. Interestingly, these results are opposite to the results obtained in the current study for the *Alpinia* rhizome, which revealed that subjecting the rhizome to microwave processing (regardless of the processing time) had a significant positive effect on TPC and antioxidant activity measured using DPPH assay. On the other hand, frying contributed to the highest decrease in antioxidant activity of the fresh rhizome, which was proved using both the DPPH and TPC methods. This can be explained by the fact that during frying there is a strong generation of free radicals as a result of the impact of high temperature on the food matrix, as well as a significant loss of antioxidants due to the conducted thermal processing [77,78,79]. In the case of the turmeric rhizome and its major active compounds, called curcuminoids, the boiling and frying process resulted in an increase in the content of these compounds (curcumin content: from 114 for the fresh rhizome to 204 mg/g after 10 min of boiling and to 224 mg/g after 10 min of frying). In the case of microwave heating, the trend was opposite—there was a decrease to 36 mg/g after 5 min [105]. The decrease in the antioxidant activity with an increase in temperature may result from ongoing processes and reactions leading to the formation of new substances with lower antioxidant activity compared to compounds present in the unprocessed raw material (for example, the formation of shogaols from gingerols in the case of ginger rhizome) [106]. A new method of *Alpinia officinarum* treatment whose effect on antioxidant activity would be worth investigating is lyophilization (freeze-drying), which uses low temperature, rather than the elevated temperature that we have used in every treatment method we have studied. The beneficial effect of lyophilization on TPC [103,107] and on the antioxidant activity determined using various methods (DPPH, FRAP, ABTS, CUPRAC) have already been investigated [103,107,108]. In contrast, in the case of *Curcuma longa*, TPC decreased after lyophilization. In addition, the antioxidant activity of turmeric as measured by DPPH and FRAP was weaker for freeze-dried turmeric extract than for fresh turmeric extract. On the other hand, in the ABTS method, the situation was reversed [109].

The thermal treatment had a variable effect on the content of active substances in the studied rhizome. Some processes, especially boiling, resulted in a significant increase in the content of active substances in the studied material. Also, a clear tendency was observed in the case of a 4-minute-long microwave heating showing an increased content of the studied components compared with the shorter processing times of 1 or 2 min. This phenomenon may occur due to the fact that a longer treatment better destroys the cellular walls and results in an increased release of the secondary metabolites. 

In turn, certain thermal processes, especially frying, contributed to the degradation of active substances and reduced their content in the studied extracts. These changes were particularly visible in more simple compounds, like the derivatives zingerone or 3-phenylpropanoic acid. The content of the latter after frying constituted less than 5% of the initial concentration. Some flavonoids were found to be more stable. Among the tested compounds, galangin was found to be the most stable compound during frying; its content decreased by ca. 50% for the first 4 min and by 66% after 10 min. For some flavonoids the content was first reduced, but later increased during the processing so as the decomposition mechanisms were less important from the increased recovery of the metabolites from the damaged cells. In the end, it needs to be underlined that based on the obtained results *Alpinia officinarum* rhizome was found to be a very good source of polyphenolic constituents with antioxidant properties and that heat treatment has a key impact on the content of secondary metabolites, the concentration of which depends mainly on the type of thermal method used as well as its duration. The research hypothesis formulated at the beginning of the research was not fully confirmed, because not all thermal processes to which the rhizome of *Alpinia officinarum* was subjected contributed to a decrease in the content of active substances. Some thermal processes—especially boiling and microwave treatment for 4 min—contributed to a significant increase in the content of the main active substances, such as pinobanksin, galangin or pinocembrin. The hypothesis that thermal processing would reduce antioxidant properties of the rhizome was definitely not confirmed, as the majority of the performed thermal treatment processes resulted in a significant increase in this activity which is in line with the information obtained for the compositional changes of single metabolites analyzed in the study.

## 4. Materials and Methods

### 4.1. Plant Material

**Figure 3:**
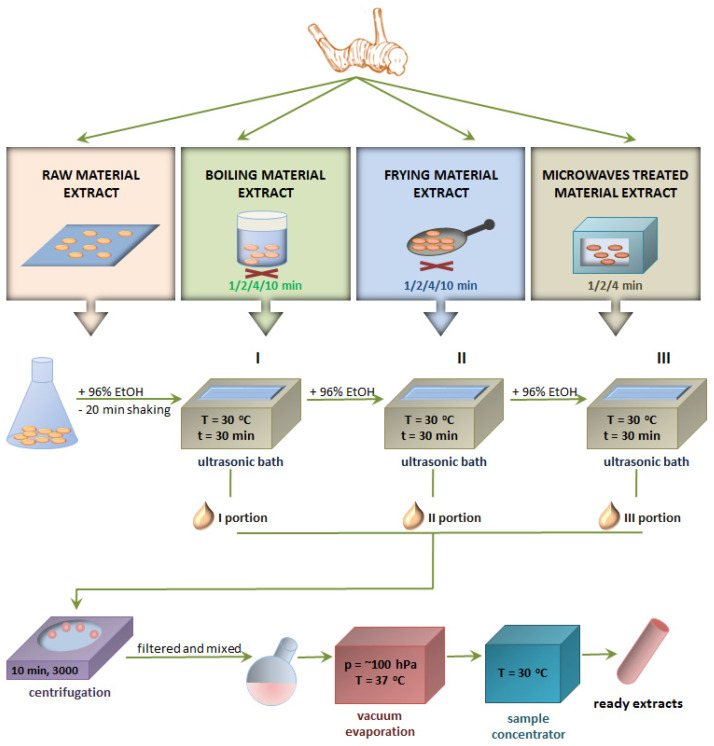
Scheme of obtaining the extract from the *Alpinia officinarum* rhizome.

The material used in the research was the rhizome of *Alpinia officinarum* cultivated in Thailand, which was bought in a certified shop for natural products in Poland. Samples of plant raw material were subjected to three thermal treatment processes: boiling, frying and microwave exposure for various durations of time (1, 2, 4 and 10 min). Fresh plant raw material not subjected to any thermal treatment was used as a comparative material. Then, the extracts were obtained from all samples and used for further tests. A scheme illustrating all activities performed during the research is presented below (Figure 3).

### 4.2. Scientific Equipment

The investigated rhizome of *Alpinia officinarum* and all reagents were weighed on AXIS AGN200 (Gdansk, Poland) and Radwag WPS 110/C/1 (Radom, Poland) scales. During the extraction process, an INFORS AG shaker (Bottmingen, Switzerland) and the EMAG EMMI 55 ultrasonic bath (Juszczyn, Poland) were used. The following were also used: to filter the extracts—the SBS-LZ-4008 electric centrifuge, Steinberg Systems (Berlin, Germany) and tissue paper filters type 388 (Munktell, Falun, Sweden), and to concentrate them—the INGOS RVO400 vacuum evaporator (Prague, Czech Republic) and Eppendorf Concentrator Plus (Warsaw, Poland). The prepared extracts were mixed with the solvent (methanol) using a VELP Vortex ZX4 shaker with an infrared sensor (Usmate, Italy). Before chromatographic analysis, all samples were filtered through a syringe filter (CronusFilters, Ø pores 0.22 µm). For chromatographic studies, the following devices were used: in the qualitative analysis—the HPLC-ESI-QTOF-MS/MS (High Performance Liquid Chromatography-Electrospray Ionization-Quadrupole Time of Flight-Mass Spectrometry) platform (Agilent Technologies, Santa Clara, CA, USA) with the Zorbax Eclipse Plus RP-18 column (d_p_ = 3.5 µm; 2.1 mm × 150 mm), in the quantitative analysis—LC-2030C 3D Plus liquid chromatograph coupled with a UV-Vis-DAD detector (Shimadzu, Japan) and a Zorbax Eclipse Plus RP-C18 column (d_p_ = 3.5 µm; 2.1 mm × 150 mm) (Agilent Technologies, Santa Clara, CA, USA), and for spectrophotometric tests—UV-Vis EVO300 PC spectrophotometer, Thermo Fisher (Waltham, MA, USA). The system for qualitative determinations of the extract also included: a degasser (G1322A), an autosampler (G1329B), a binary pump (G1312C), a photodiode detector—DAD (G1315D) and a mass spectrometer (G6530B). To acquire the MS spectra and process the obtained data, Mass Hunter Workstation software (Agilent Technologies, Santa Clara, CA, USA, B.10.00 version) was used.

### 4.3. Chemicals

All chemicals and reagents used in the study were of analytical purity. Methanol, ethanol and sodium carbonate (Na_2_CO_3_) were obtained from Avantor Chemicals (Gliwice, Poland). Ultrapure water (resistance 18.2 MΩcm) used during all determinations was obtained using an Ultrapure Millipore Direct-Q-R 3UV (Millipore, Bedford, MA, USA). The standard substances for HPLC analysis—6-gingerol and kaempferol, acacetin and galangin which were used for a direct comparison of fragmentation patterns in the LC-MS method (≥98% HPLC) and for UV-Vis spectrophotometry—gallic acid, Folin–Ciocalteu reagent and 2,2-diphenyl-1-1-picrylhydrazyl radical (DPPH) were purchased from Sigma Aldrich (St. Louis, MO, USA). The mobile phase in HPLC was composed of HPLC-grade water [solvent A] purchased from ChemSolve (Łódź, Poland), HPLC-grade acetonitrile (ACN) [solvent B] and formic acid (HCOOH) purchased from J.T. Baker (Phillipsburg, NJ, USA).

### 4.4. Thermal Treatment

#### 4.4.1. Fresh Rhizome Extract

The fresh raw rhizome was cut into small pieces and samples of 50 g were weighed into dry conical flasks. To the content of each flask was added 70 mL of 96% ethanol. The flasks were then placed on an electric shaker at 250 RPM for 20 min of maceration. Next, the flasks were transferred to an ultrasonic bath filled with distilled water at 30 °C for 30 min. The extraction process in the ultrasonic bath was repeated three times, each time pouring off the extract and adding a new portion of ethanol. In the result, the ratio of ethanol used for extraction to raw material was 1:1.4. Later, the obtained fluid extract was poured in equal volumes into test tubes and placed in a centrifuge. The samples were centrifuged for 10 min at a speed of 3000 RPM. After centrifugation, each sample was filtered through a clean paper filter type 388 (Chemland, Stargard, Poland) into a round-bottom flask. The flasks were placed on an evaporator to evaporate the solvent under reduced pressure at a low temperature (T < 37 °C). The excess residual solvent was evaporated using an Eppendorf Concentrator Plus.

#### 4.4.2. Boiling

The plant material was cut into slices and then four portions (~25 g) were weighed. Using a cylinder, 250 mL of distilled water was measured and then poured into four clean 400 mL beakers. Then, the raw material was placed in beakers with already boiling water for 1, 2, 4 and 10 min. After the designated time, the material was removed from the water, cut into small cubes and extracted using the procedure described above.

#### 4.4.3. Frying

Four portions of plant material (~25 g) were weighed successively. They were then fried in a heated pan without oil for 1, 2, 4 and 10 min, turning them over halfway through. In the next step, thermally processed plant material was extracted as mentioned above.

#### 4.4.4. Microwave Processing

The raw material cut into slices was weighed (~25 g) and then heated in a microwave oven with a power of 800 W for 1, 2 and 4 min. Microwave treatment for 10 min carbonized the samples and therefore these were not included in further studies. Thermally processed samples were then extracted in a similar way. 

Extraction yield for all extracts was presented in Table S2 in the Supplementary Materials. 

### 4.5. Chromatographic Analysis of the Studied Material

#### 4.5.1. HPLC-ESI-QTOF-MS/MS Fingerprinting of the Extract

For qualitative purposes, the samples were analyzed for their fingerprint and composition using the HPLC-ESI-QTOF-MS/MS platform according to previously described procedure with further modifications [110]. Prior to the analysis, the extracts were dissolved in methanol (at a concentration of 10 mg/mL) and then filtered through a nylon syringe filter with pore size of 0.1 µm. The following gradient elution mode was used: eluent A: ultrapure water; eluent B: acetonitrile; both phases contained formic acid at a concentration of 0.1%. A single analysis lasted 45 min and the mobile phase composition (solvent A/solvent B) in time was as follows: 0 min—99%/1%, 3 min—80%/20%, 30 min—25%/75%, 36 min—5%/95%, 38.5 min—80%/20% and 45 min—the end of analysis. The injection volume was 5 μL at a solvent flow rate of 0.2 mL/min. The operating range of the DAD detector was 210–320 nm. Collected spectra were scanned in the *m/z* range 100–1700 Da in the negative ionization mode. The other analysis parameters were as follows: gas and thermostat temperatures—275 °C and 25 °C; gas flow—12 L/min; voltage of the skimmer, fragmentor and capillary—65, 110 and 3000 V; pressure of nebulizer—35 psig; collision energy—10 and 20 V.

#### 4.5.2. HPLC-Based Quantitative Analysis

The same extract solution (described above) was used for the quantitative study using HPLC analysis according to previously described procedure with further modifications [110]. During analysis solvent gradient elution mode was used. The mobile phase was composed of eluent A: ultrapure water and eluent B: acetonitrile; both phases were enriched with formic acid at the concentration of 0.1%. A single analysis lasted 45 min and the mobile phase composition (solvent A/solvent B) in time was as follows: 0 min—99%/1%, 3 min—80%/20%, 30 min—25%/75%, 36 min—5%/95%, 38.5 min—80%/20% and 45 min—the end of analysis. The settings of the chromatograph were as follows: flow rate of 0.2 mL/min; injection volume of 5 µL; a thermostat temperature of 25.0 °C; UV detection range of 190–500 nm; and DAD detection wavelength of 290 nm. 6-gingerol standard solution (10 mg/mL in methanol) was used for quantitative analysis. Based on the obtained determinations, the calibration curve was plotted, which was characterized by the following equation: y = 5,439,294.167x + 83,057.91667 (R^2^ = 0.999) and linearity in the range of 3–100 mg/kg. The recovery determined for the standard was equal to 95% (*n* = 5). The repeatability of the quantitative determinations was higher than 90%. The limit of detection (LOD) value expressed as signal-to-noise (S/N) times three for the investigated standard in the prepared method was calculated to be 0.34 mg/kg (*n* = 5), whereas the limit of quantification (LOQ) calculated as S/N times 10 was 1.12 mg/kg (*n* = 5).

#### 4.5.3. Spectrophotometric Determinations of the Antioxidant Activity of the Studied Extracts

##### Free-Radical Scavenging Activity (DPPH Test)

For the determinations using DPPH radical, an original protocol with further modifications was used [111,112]. An amount of 100 µL of each extract at the concentration of 10 mg/mL in methanol was mixed with 3.9 mL of freshly prepared methanol solution of the DPPH radical (with absorbance at a wavelength of 517 nm~0.9). The contents of the test tubes were mixed thoroughly using a shaker. In addition to the studied samples, a solution with the addition of 100 µL of gallic acid solution with a concentration of 50 mg/mL in methanol was used as a reference sample. All samples were incubated at room temperature in the dark for 30 min. After the time had elapsed, the absorbance was measured using a spectrophotometer for each sample at a wavelength of 517 nm. Results were calculated using the following formula:

(1) $$\%inhibition=\left[\frac{A_{0}-A}{A_{0}}\right]\times 100\%$$

where: *A*_0—_the initial absorbance of the DPPH, *A*—absorbance of the DPPH solution at the end of the reaction.

##### Determination of Total Phenolic Content (TPC)

The same extracts from fresh and thermally processed *Alpinia officinarum* were used to determine the TPC value of the samples according to previously described procedure with further modifications [112,113]. For this purpose, 100 µL of extract was mixed with 6 mL of ultrapure water, 0.5 mL of Folin–Ciocalteu reagent and 1.5 mL of 20% sodium carbonate water solution. Each test tube was filled with ultrapure water to a final volume of 10 mL. To plot the calibration curve, standard solutions of gallic acid in the concentration range of 50–1000 mg/L were prepared, added instead of the studied samples and analyzed simultaneously using the same procedure. All samples were incubated at room temperature in the dark for 2 h. After this time, the solution was mixed and the absorbance was determined in 1 cm cuvettes at a wavelength of 760 nm. Using the prepared calibration curve, the total content of polyphenolic compounds in the studied samples was read, calculated and expressed as gallic acid equivalents (mg GAE/L) (standard solution). 

### 4.6. Statistical Analysis

All analyses were performed in three replicates. The final results are presented as mean ± standard deviation (SD). Differences between the groups were analyzed with a two-way ANOVA (one qualitative factor and repeatable measurements) followed by a Tukey’s post hoc test. Dunnett’s post hoc test was applied to compare the results obtained to the control sample. Pearson correlations were used to assess the relationship between compound content and antioxidant activity. Results were considered statistically significant when *p* < 0.05. Statistical analysis was performed using Statistica Software (v13.3, StatSoft, Kraków, Poland).

## 5. Conclusions

During the conducted research, eight compounds were identified and quantified in the *Alpinia officinarum* rhizome: zingerone, 3-phenylpropanoic acid, pinobanksin, kaempferol, pinocembrin, galangina, kaempferide and acacetin. Pinobanksin was determined in the highest amounts, whereas pinocembrin was found in the lowest concentration. In general, thermal processing reduced the content of active constituents in the rhizome, but there were some exceptions to this trend. Boiling for 4 min had a positive impact on the content of various active constituents present in the rhizome. Moreover, it was proved that microwave processing for 4 min had a positive influence on the content of pinocembrin. It was also proved that thermal processing may have a positive impact on antioxidant properties of the rhizome of *Alpinia officinarum*. The TPC method using Folin–Ciocalteu reagent showed that the raw material exposed to microwaves for 4 min had the highest antioxidant activity compared to other thermal treatment methods in various time units. The lowest TPC values were determined in the material fried for 10 min (94.28 mg/L). In both the TPC and DPPH tests, frying turned out to be the only process that caused a decrease in the antioxidant activity of the extracts (especially for longer treatment times). Thermal treatment has a significant impact on the content of the studied polyphenolic compounds, probably as a result of its degradation in higher temperature. From all investigated thermal processing methods, it seems that frying has the most detrimental effect towards *Alpinia officinarum* rhizome regarding its composition and antioxidant activity, and this process seems to be the most adverse when using this plant as a food ingredient. 

## Figures and Tables

**Table 1:** The identified compounds present in *Alpinia officinarum* rhizome extract in negative ion mode (RDB—number of double bonds and rings; Error—error of mass measurement).

Proposed Compound	Zingerone	3-Phenylpropanoic Acid	Pinobanksin	Kaempferol	Pinocembrin	Galangin	Kaempferide	Acacetin
Molecular formula	C_11_H_14_O_3_	C_9_H_10_O_2_	C_15_H_12_O_5_	C_15_H_10_O_6_	C_15_H_12_O_4_	C_15_H_10_O_5_	C_16_H_12_O_6_	C_16_H_12_O_5_
Retention time [min]	10.01	12.3	15.0	16.4	22.4	22.7	22.9	23.8
Theoretical *m/z*	193.0870	149.0608	271.0612	285.0405	255.0663	269.0455	299.0561	283.0612
Measured *m/z*	193.0870	149.0609	271.0612	285.0413	255.0665	269.0459	299.0558	283.0611
Error [ppm]	0.09	–0.65	–0.01	–2.93	–0.85	–1.3	1.04	0.34
RDB	5	5	10	11	10	11	11	11
Collision energy [V]	10	10	20	20	20	20	20	10
MS/MS fragment ions with signal intensity%	179.0030 (8.17%)	105.0556 (16.31%)	253.0509 (35%), 225.0563 (7.11%), 197.0618 (15.24%) 151.0041 (14.21%) 125.0247 (17.16%)	257.0453 (1.86%) 229.0495 (1.79%)	213.0594 (26.34%) 151.0004 (24.7%) 145.0650 (11.05%)	227.0360 (1.22%) 213.0564 (2.22%) 197.0619 (1.79%)	284.0318 (100%) 164.0104 (1.73%) 151.0029 (7.14%)	268.0391(100%) 239.0359 (96.2%)

**Table 2:** The influence of various thermal processes on the content of active substances identified in the rhizome of *Alpinia officinarum*.

	Time (min)			
	1 min	2 min	4 min	10 min
Zingerone (mg/kg)				
Fresh rhizome	185.48 ± 0.99			
Boiling	104.65 ± 3.00 *** ^1 a^	89.04 ± 7.41 *** ^2 a^	97.07 ± 1.30 *** ^12 a^	56.16 ± 1.87 *** ^3 a^
Frying	96.13 ± 2.44 *** ^1 a^	80.24 ± 1.07 *** ^23 a^	76.64 ± 0.93 *** ^3 b^	82.86 ± 0.94 *** ^2 b^
Microwaves	107.01 ± 1.21 *** ^1 b^	82.71 ± 0.93 *** ^2 a^	188.59 ± 1.18 ^3 c^	
3-Phenylpropanoic acid (mg/kg)				
Fresh rhizome	81.59 ± 3.00			
Boiling	41.5 ± 1.52 *** ^1 a^	80.39 ± 4.09 ^2 a^	19.29 ± 0.15 *** ^3 a^	15.8 ± 0.46 *** ^3 a^
Frying	6.05 ± 0.09 *** ^1 b^	4.55 ± 0.48 *** ^2 b^	3.49 ± 0.06 *** ^3 b^	5.9 ± 0.22 *** ^1 b^
Microwaves	13.64 ± 0.73 *** ^1 c^	7.12 ± 0.04 *** ^2 b^	87.64 ± 1.78 ** ^3 c^	
Pinobanksin (mg/kg)				
Fresh rhizome	537.79 ± 21.18			
Boiling	580.99 ± 3.36 *** ^1 a^	503.16 ± 2.37 *** ^2 a^	1162.4 ± 7.39 *** ^3 a^	697.42 ± 2.80 *** ^4 a^
Frying	44.27 ± 0.23 *** ^1 b^	119.15 ± 3.93 ** ^2 b^	232.4 ± 4.07 *** ^3 b^	294.08 ± 0.82 *** ^4 b^
Microwaves	379.77 ± 1.07 *** ^1 c^	293.53 ± 0.82 *** ^2 c^	216.05 ± 2.95 *** ^3 c^	
Kaempferol (mg/kg)				
Fresh rhizome	64.53 ± 5.56			
Boiling	85.56 ± 1.82 *** ^1 a^	78.09 ± 1.29 *** ^2 a^	79.41 ± 1.26 *** ^2 a^	19.52 ± 0.23 *** ^3 a^
Frying	16.95 ± 0.38 *** ^1 b^	20.45 ± 1.17 ** ^2 b^	12.93 ± 0.23 *** ^3 b^	13.82 ± 0.07 *** ^3 b^
Microwaves	17.84 ± 0.09 *** ^b^	13.79 ± 0.07 *** ^c^	39.33 ± 2.43 *** ^c^	
Pinocembrin (mg/kg)				
Fresh rhizome	21.51 ± 0.03			
Boiling	26.48 ± 0.41 ^1 a^	10.24 ± 0.17 *** ^2 a^	31.31 ± 0.18 *** ^3 a^	3.72 ± 0.03 *** ^4 a^
Frying	28.34 ± 2.62 *** ^1 a^	49.59 ± 1.53 *** ^2 b^	62.89 ± 4.32 *** ^3 b^	89.83 ± 2.19 *** ^4 b^
Microwaves	116.01 ± 2.83 *** ^1 b^	89.66 ± 2.19 *** ^2 c^	212.94 ± 5.38 *** ^3 c^	
Galangin (mg/kg)				
Fresh rhizome	197.67 ± 0.09			
Boiling	157.23 ± 0.61 *** ^1 a^	78.67 ± 0.42 *** ^2 a^	280.69 ± 2.21 *** ^3 a^	146.61 ± 0.39 *** ^4 a^
Frying	85.28 ± 4.6 *** ^12 b^	95.7 ± 1.93 *** ^2 b^	81.67 ± 7.21 *** ^3 b^	68.22 ± 2.86 *** ^4 b^
Microwaves	88.1 ± 3.70 *** ^1 b^	68.1 ± 2.86 *** ^2 c^	109.14 ± 6.88 *** ^3 c^	
Kaempferide (mg/kg)				
Fresh rhizome	58.25 ± 0.82			
Boiling	62.43 ± 0.42 * ^1 a^	37.13 ± 0.12 *** ^2 a^	63.72 ± 0.29 ** ^3 a^	12.21 ± 0.14 *** ^4 a^
Frying	7.93 ± 1.48 *** ^1 b^	15.3 ± 0.47 *** ^2 b^	3.55 ± 0.31 *** ^3 b^	7.62 ± 0.65 *** ^1 b^
Microwaves	9.61 ± 0.45 *** ^1 b^	7.6 ± 0.65 *** ^1 c^	79.22 ± 5.57 *** ^c^	
Acacetin (mg/kg)				
Fresh rhizome	82.68 ± 0.62			
Boiling	57.42 ± 0.12 *** ^1 a^	10.91 ± 0.09 *** ^2 a^	29.52 ± 0.42 *** ^3 a^	20.59 ± 0.36 *** ^4 a^
Frying	22.5 ± 1.66 *** ^1 b^	31.42 ± 0.68 *** ^2 b^	12.55 ± 0.41 *** ^3 b^	21.02 ± 1.39 *** ^1 a^
Microwaves	27.01 ± 1.56 *** ^1 c^	20.98 ± 1.38 *** ^2 c^	30.94 ± 0.47 *** ^3 c^	

Data in the table: means ± SD; *n* = 3. Comparison to control (fresh rhizome); Dunnet’s test; * *p* < 0.05; ** *p* < 0.01; ***p < 0.001. The means not sharing the same letter in a column are significantly different at *p* < 0.05; comparisons separately for each compound and separately for each time point; Tukey test. Means not sharing the same superscript number in a row are significantly different at *p* < 0.05; comparisons separately for each compound and separately for each type of treatment; Tukey test.

**Table 3:** Antioxidant activity of thermally processed rhizome of *Alpinia officinarum*.

	Time (min)			
	1 min	2 min	4 min	10 min
DPPH (% inhibition)				
Fresh rhizome	27.28 ± 4.25			
Boiling	80.98 ± 0.84 *** ^1 a^	78.83 ± 3.57 *** ^1 a^	71.14 ± 4.35 *** ^2 a^	81.78 ± 1.29 *** ^1 a^
Frying	31.75 ± 3.18 ^1 b^	34.21 ± 1.40 ** ^1 b^	21.60 ± 1.00 * ^2 b^	24.39 ± 1.67 ^2 b^
Microwaves	79.34 ± 3.16 *** ^1 a^	50.99 ± 5.39 *** ^2 c^	50.44 ± 3.20 *** ^2 c^	
TPC (mg GAE/L)				
Fresh rhizome	128.32 ± 7.7			
Boiling	266.19 ± 25.89 *** ^12 a^	286.54 ± 12.35 *** ^2 a^	222.09 ± 10.18 *** ^3 a^	252.24 ± 25.59 *** ^13 a^
Frying	109.49 ± 10.02 ^1 b^	162.44 ± 10.66 ** ^2 b^	100.74 ± 10.55 * ^13 b^	94.28 ± 2.61 ** ^3 b^
Microwaves	227.2 ± 17.64 *** ^1 c^	190.07 ± 17.1 *** ^2 c^	323.15 ± 20.46 *** ^3 c^	

Data in table: Mean ± SD; *n* = 6. Comparison to control (Fresh rhizome); Dunnet’s post hoc test; * *p* < 0.05; ** *p* < 0.01; *** *p* < 0.001. Averages not having the same letter in a column are statistically significantly different at *p* < 0.05; Tukey’s post hoc test. Averages not having the same number in a row are statistically significantly different at *p* < 0.05; Tukey’s post hoc test.

**Table 4:** Correlation between compound content and antioxidant activity.

	Zingerone	3-Phenylpropanoic Acid	Pinobanksin	Kaempferol	Pinocembrin	Galangin	Kaempferide	Acacetin
TPC (mg GAE/L)	0.21	0.54 ***	0.36 *	0.47 **	0.24	0.19	0.57 ***	−0.03
DPPH (% inhibition)	−0.14	0.23	0.61 ***	0.51 **	−0.18	0.32	0.32	−0.07

Pearson correlations: * *p* < 0.05; ** *p* < 0.01; ***p < 0.001.

## Data Availability

Data are contained within the article or supplementary material.

## Supplementary Materials

The following supporting information can be downloaded at: https://www.mdpi.com/article/10.3390/ijms25073625/s1.
