# Supplementary material for: Influence of Thermal Treatment on the Composition of Alpinia officinarum Rhizome
Source: Int J Mol Sci. 2024 Mar 24;25(7):3625. doi: 10.3390/ijms25073625 (PMC11012154; doi:10.3390/ijms25073625)
Supplement: Supplementary file 1 [file ijms-25-03625-s001.zip › ijms-2911018-supplementary.pdf]

SUPPLEMENTARY FILE

Influence of thermal treatment on the composition of *Alpinia officinarum* rhizome

Justyna Zagórska [1], Karolina Pietrzak [1], Wirginia Kukula-Koch [2], Marcin Czop[3], Karolina Wojtysiak [1] and Wojciech Koch [1,*]

[1] Department of Food and Nutrition, Medical University in Lublin, 4a, Chodzki Str., 20-093 Lublin, Poland: justyna.zagorska@umlub.pl, karolinapietrzak94@gmail.com, kochw@interia.pl
[2] Department of Pharmacognosy with Medical Plants Garden, Medical University of Lublin, 1 Chodzki Str., 20-093 Lublin, Poland; virginia.kukula@gmail.com
[3] Department of Clinical Genetics, Medical University of Lublin, 11 Radziwiłłowska Str.,20-080 Lublin, Poland; marcin.czop@umlub.pl

* Correspondence: kochw@interia.pl; Tel.: +48-81-448-7143

Table S1. The MS/MS chromatograms of the identified components of *Alpinia officinarum* extract.

**1 – zingerone**	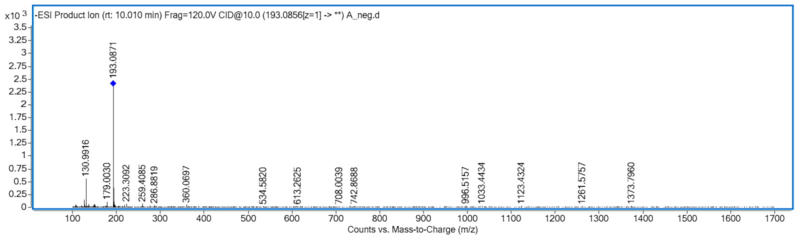
**2 – 3-phenylpropanoic acid**	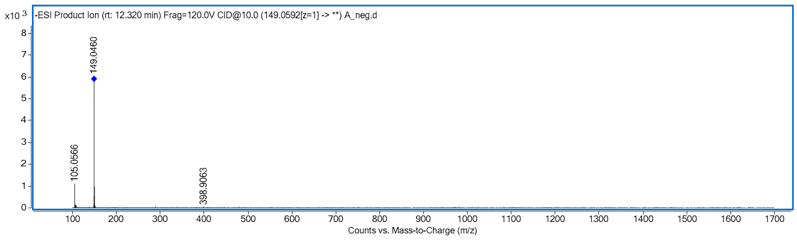
**3 – pinobanksin**	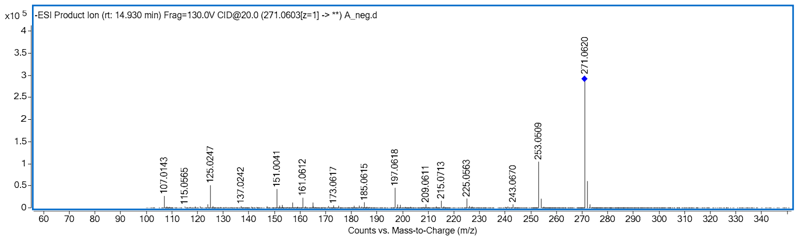
**4 – kaempferol**	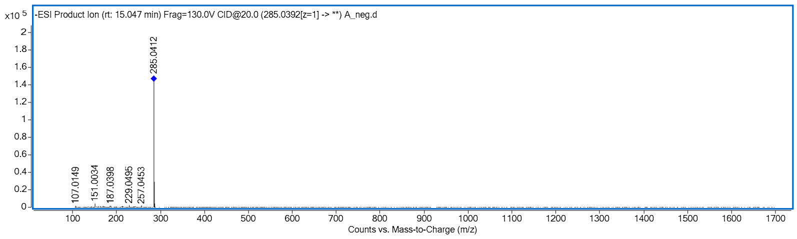

**5 – pinocembrin**	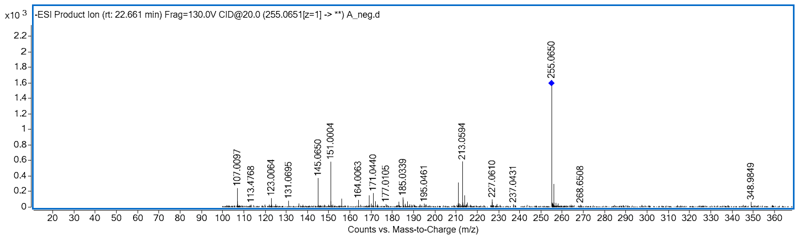
**6 – galangin**	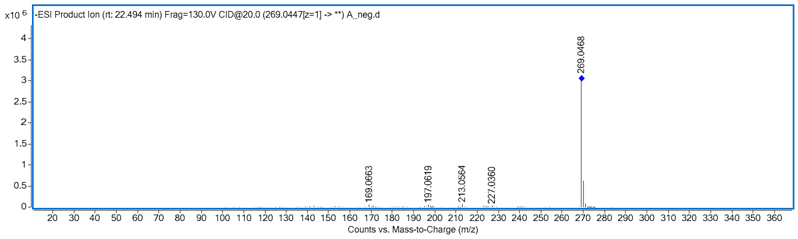
**7 – kaempferide**	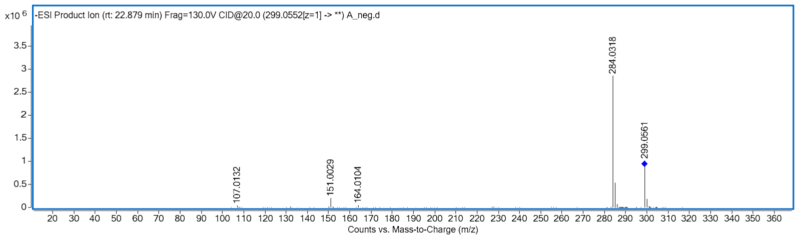
**8 – acacetin**	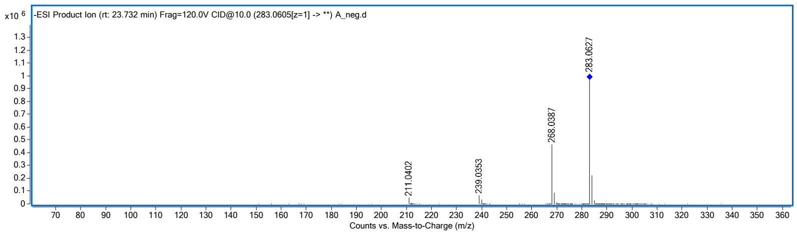

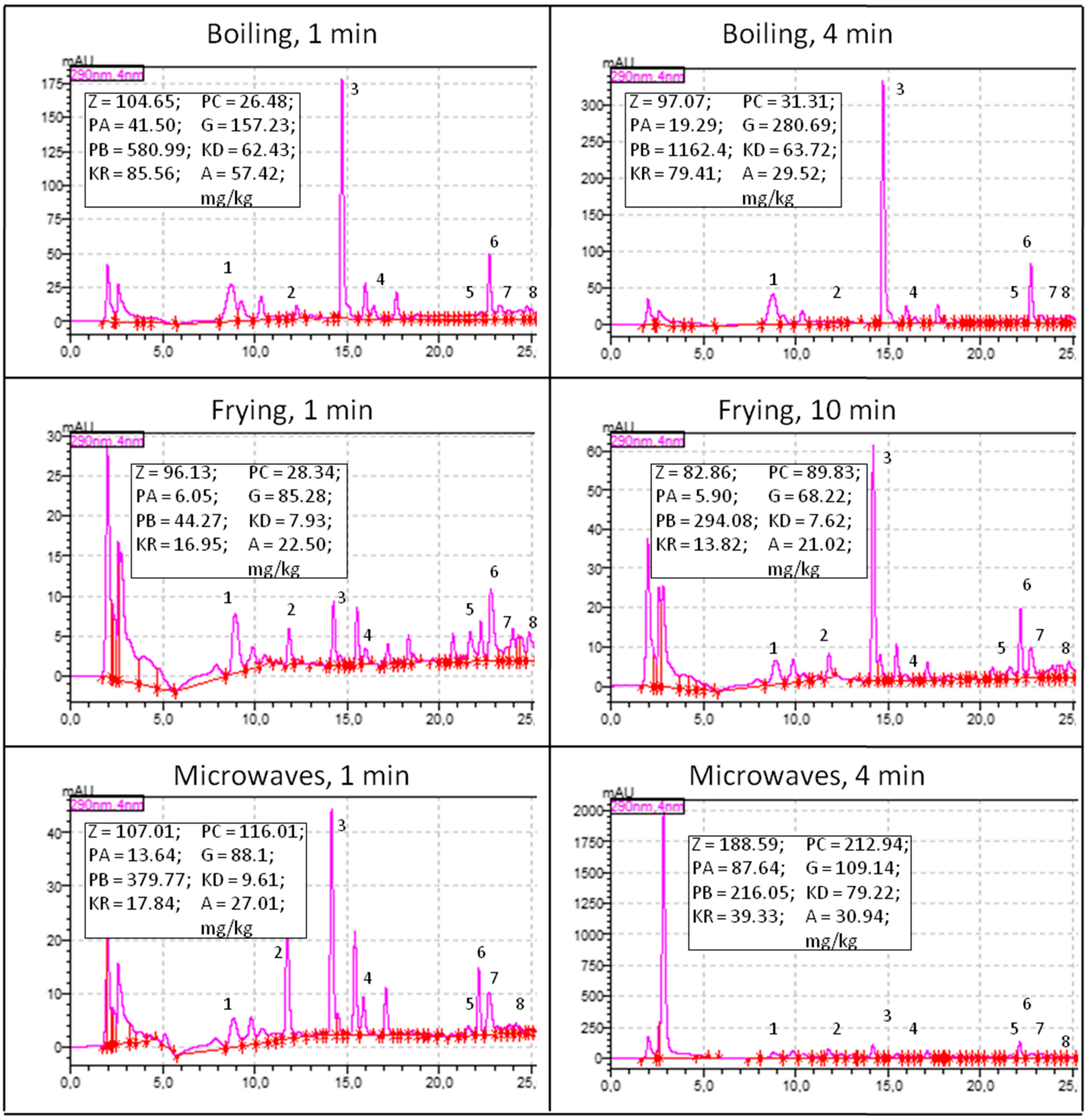

Figure S1. HPLC chromatograms recorded for the analysed samples that undergone the thermal processing by boiling, frying and microwave heating. Z – zingerone (1), PA – 3-phenylpropanoic acid (2), PB – pinobanksin (3), KR – kaempferol (4), PC – pinocembrin (5), G – galangin (6), KD – kaempferide (7), A – acacetin (8).

Table S2. Extraction yield.

Sample	Mass of raw rhizome [g]	Mass of extract [g]
**Fresh**	50.062	1.6748
**Boiling**		
1 min	23.649	0.3694
2min	24.261	0.3745
4 min	24.382	0.4107
10 min	24.482	0.2588
**Frying**		
1 min	25.259	0.7295
2 min	25.124	0.6794
4 min	25.131	0.8351
10 min	25.183	0.6285
**Microwaves**		
1 min	23.643	0.7620
2 min	24.311	0.6056
4 min	24.617	0.1916
